# Supplementary figures and images for: A Molecular Characterization of the Allelic Expression of the BRCA1 Founder Δ9–12 Pathogenic Variant and Its Potential Clinical Relevance in Hereditary Cancer
Source: Int J Mol Sci. 2024 Jun 20;25(12):6773. doi: 10.3390/ijms25126773 (PMC11204022; doi:10.3390/ijms25126773)

## Figure S1

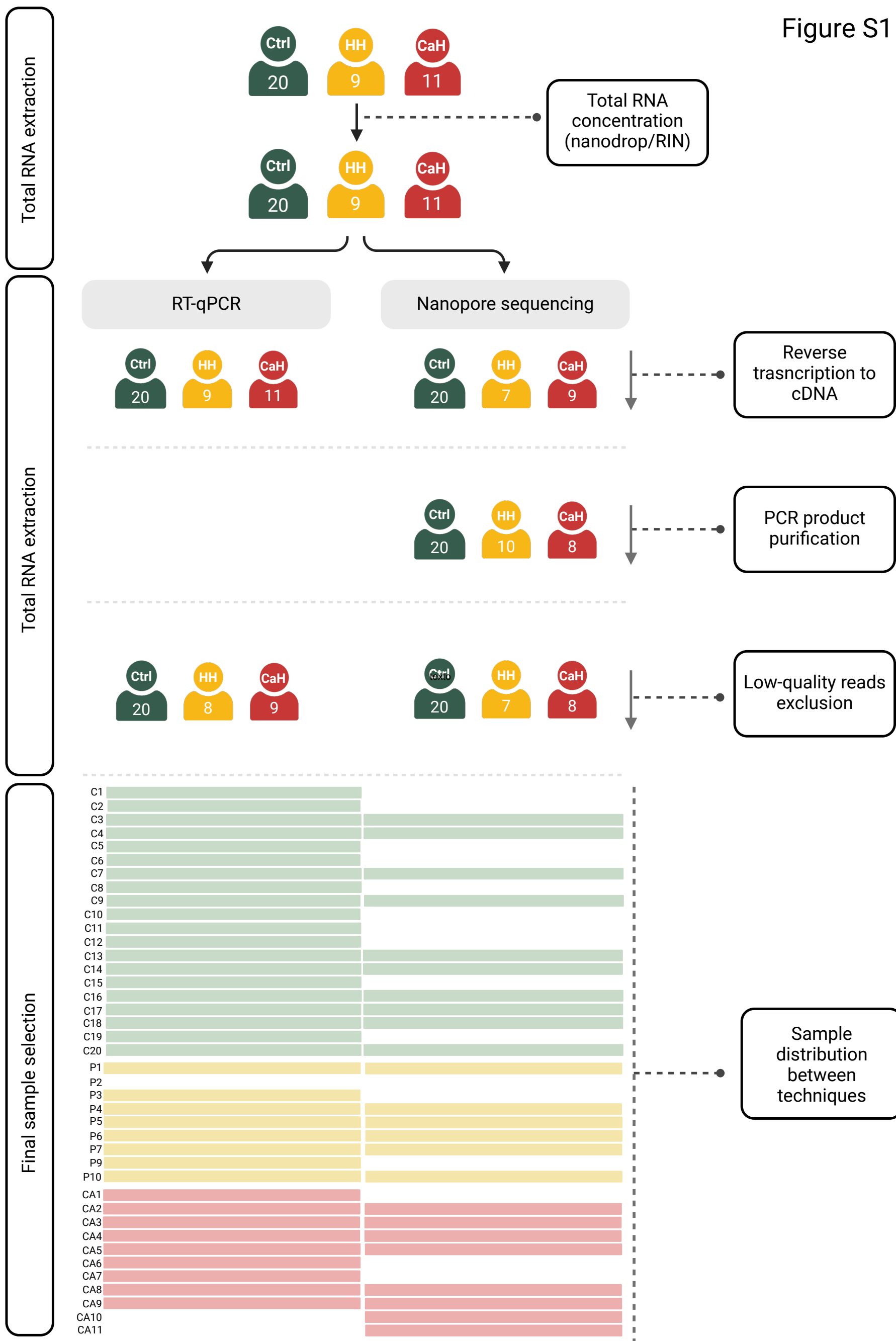

Figure S2

A

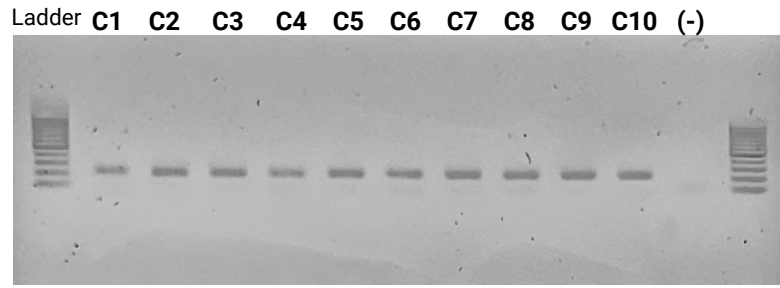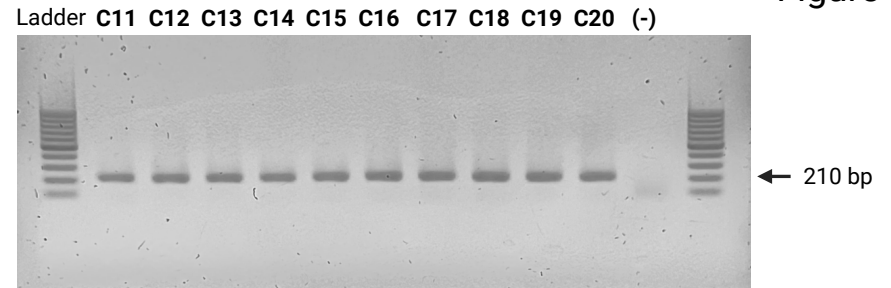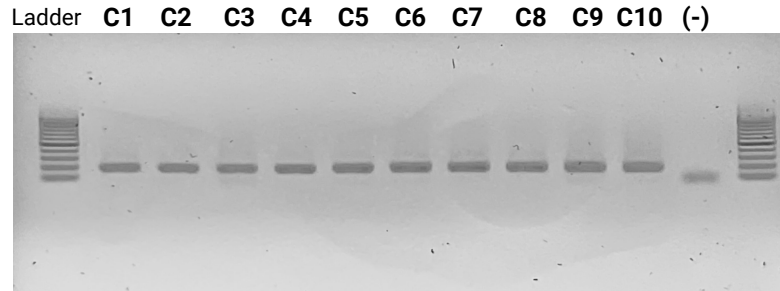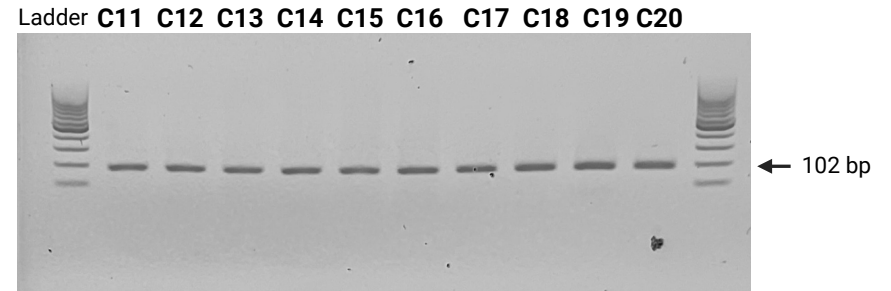

B

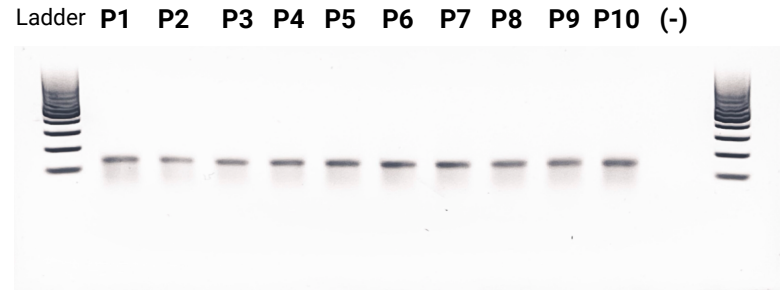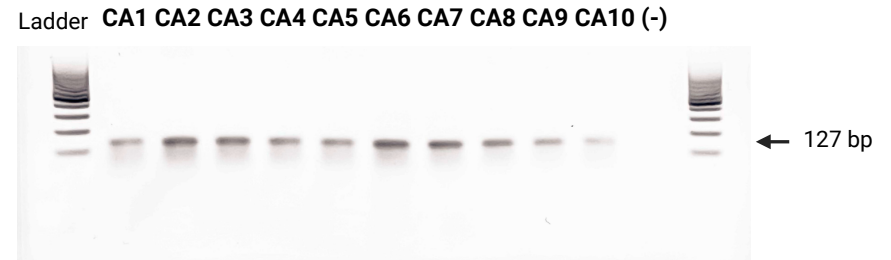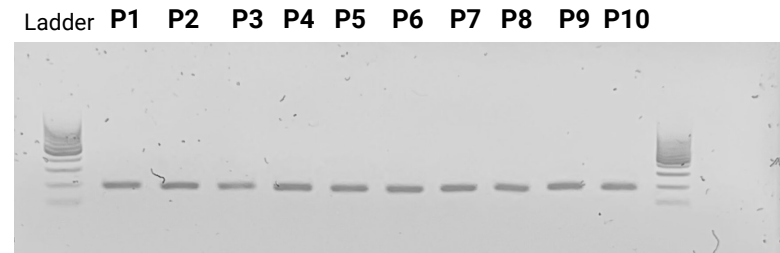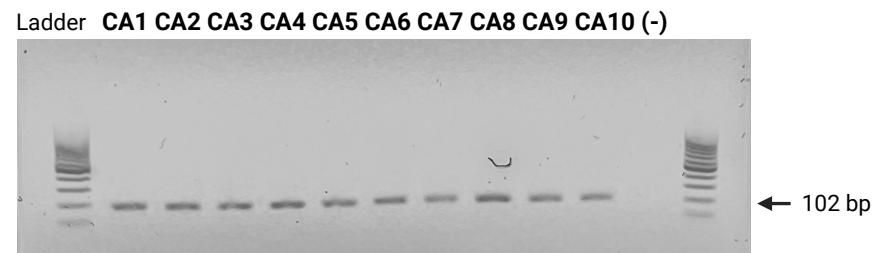

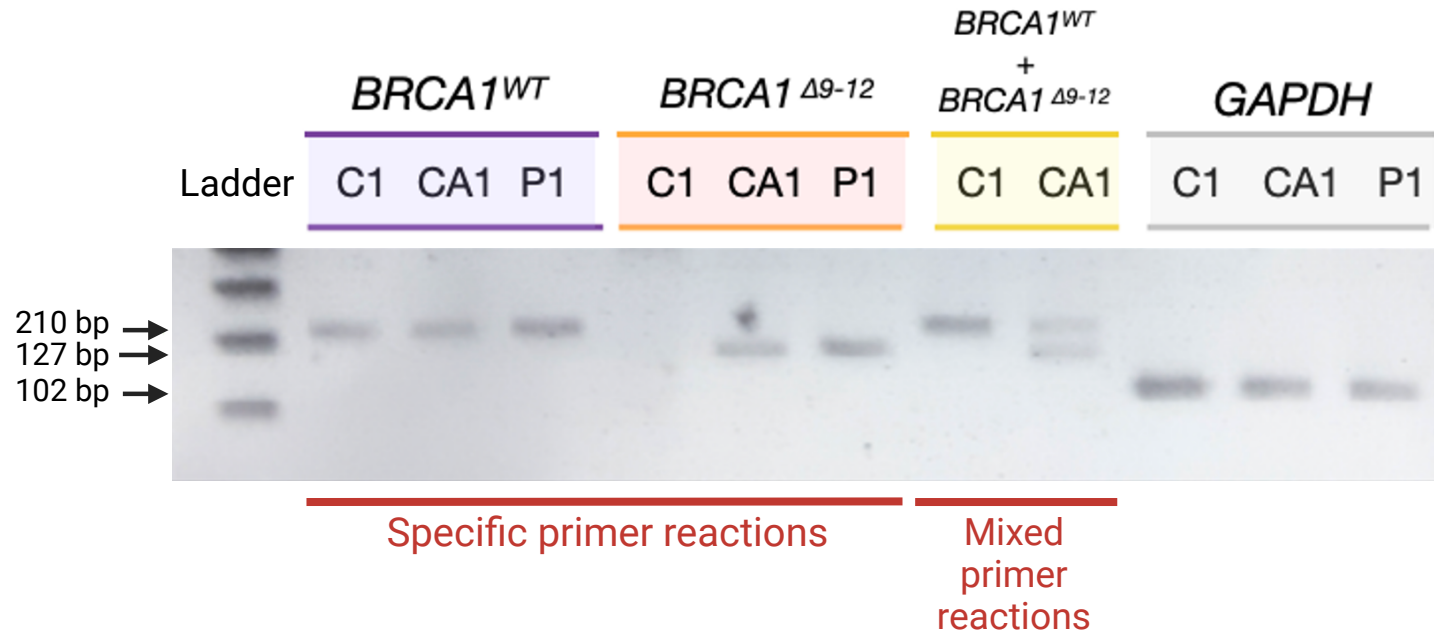

Figure S4

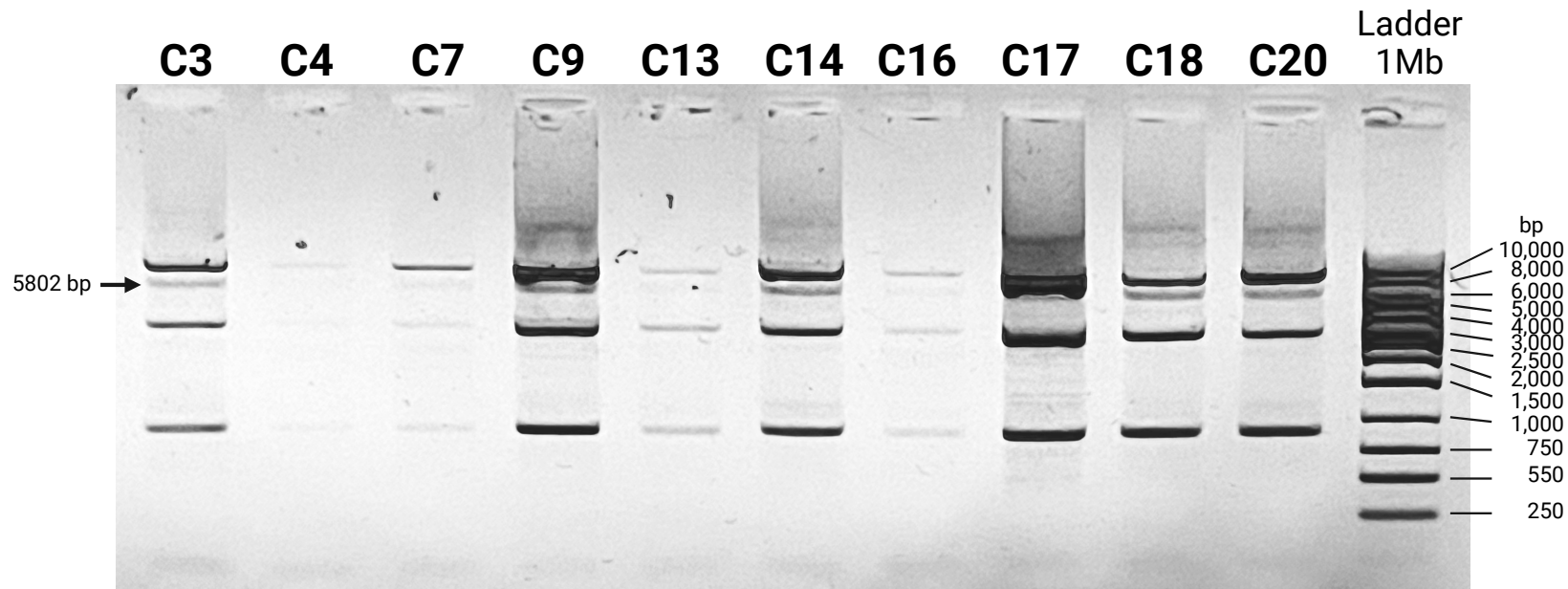

Supplement: Supplementary file 1 [file ijms-25-06773-s001.zip › ijms-3005379-supplementary.pdf]
